# Luminescence Thermometry with Eu^3+^-Doped Y_2_Mo_3_O_12_: Comparison of Performance of Intensity Ratio and Machine Learning Temperature Read-Outs

**DOI:** 10.3390/ma17215354

**Published:** 2024-11-01

**Authors:** Tamara Gavrilović, Vesna Đorđević, Jovana Periša, Mina Medić, Zoran Ristić, Aleksandar Ćirić, Željka Antić, Miroslav D. Dramićanin

**Affiliations:** Center of Excellence for Photoconversion, Vinča Institute of Nuclear Sciences—National Institute of the Republic of Serbia, University of Belgrade, P.O. Box 522, 11001 Belgrade, Serbia; jburojevic@vinca.rs (J.P.); mina@vin.bg.ac.rs (M.M.); risticz@vin.bg.ac.rs (Z.R.); aleksandar.ciric@ff.bg.ac.rs (A.Ć.); zeljka.antic@vin.bg.ac.rs (Ž.A.); dramican@vin.bg.ac.rs (M.D.D.)

**Keywords:** luminescence thermometry, luminescence intensity ratio, principal component analysis, europium, Y_2_Mo_3_O_12_

## Abstract

Accurate temperature measurement is critical across various scientific and industrial applications, necessitating advancements in thermometry techniques. This study explores luminescence thermometry, specifically utilizing machine learning methodologies to enhance temperature sensitivity and accuracy. We investigate the performance of principal component analysis (PCA) on the Eu^3+^-doped Y_2_Mo_3_O_12_ luminescent probe, contrasting it with the traditional luminescence intensity ratio (LIR) method. By employing PCA to analyze the full emission spectra collected at varying temperatures, we achieve an average accuracy (ΔT) of 0.9 K and a resolution (δT) of 1.0 K, significantly outperforming the LIR method, which yielded an average accuracy of 2.3 K and a resolution of 2.9 K. Our findings demonstrate that while the LIR method offers a maximum sensitivity (Sr) of 5‰ K⁻^1^ at 472 K, PCA’s systematic approach enhances the reliability of temperature measurements, marking a crucial advancement in luminescence thermometry. This innovative approach not only enriches the dataset analysis but also sets a new standard for temperature measurement precision.

## 1. Introduction

The accurate and precise measurement of temperature is essential for industrial applications and scientific research. Many physical properties, including specific heat, thermal conductivity, coefficient of thermal expansion, elastic modulus, yield strength, stiffness, diffusion, and flow stress are influenced by temperature and are often measured under varying thermal conditions [1,2,3,4]. Thermometry, the practice of measuring temperature, is performed using a wide range of instruments and methods. Common temperature sensing techniques include liquid-in-glass thermometers, thermocouples, pyrometers, and thermometers based on gas expansion, manometric principles, resistance, semiconductors, fiber optics, quartz, and ultrasonic technologies [5]. However, there is an immediate demand for noncontact thermometry for moving items that are sensitive to contact, difficult to reach, or in hazardous environments. To meet the needs, temperature measurements that take advantage of changes in material optical characteristics are regarded as promising. Optical methods of interest include Raman and Rayleigh scattering, near-field scanning optical microscopy, thermoreflectance, optical interferometry, and luminescence spectroscopy [3,6,7,8,9]. Regarding the temperature dependence of the luminescence properties of materials, luminescence thermometry has gathered the most attention owing to its multiple qualities such as high resolution and precision, quick response, and non-invasiveness [1,10,11,12].

Luminescent thermometry is based on temperature-induced changes in the luminescent properties of temperature probes. Parameters, such as the spectral positions of emission and excitation bands, emission peak intensity or width, the shape of the emission spectra, polarization, excited state lifetimes, and rise times can be used for measuring temperature. They can be used separately or in combination to perform single-parameter or multiparametric thermometry [13,14].

Numerous luminescent temperature probes and readouts were reported in recent years [14,15,16,17,18,19]. In addition to fundamental breakthroughs, temperature sensor probes found use in distinct applications fields: metrology, aerodynamics, climate research, medicine, military technology, biomedical, air conditioning, food storage, and other goods, mining research, etc. [1,3,20,21,22,23,24,25,26,27]. The development of innovative luminescent probes or the refinement and expansion of theoretical frameworks may not be sufficient to meet current research and development needs in emerging industries and novel scientific investigations [28]. Improving the methodologies and technology used to evaluate luminescence data could be achieved by employing innovative methods in temperature read-outs. Potential innovations are creating more precise, real-time, or user-friendly readout systems, incorporating new detection technologies, or improving signal processing procedures.

As a part of artificial intelligence, machine learning is focused on developing algorithms that allow computers to learn from and make data-based decisions. Machine learning models can automatize tasks ranging from image recognition and natural language processing to medical diagnosis and agriculture by analyzing patterns and iteratively developing their performance [29,30,31,32]. Techniques such as supervised and unsupervised learning and reinforcement learning drive advancements in robotics, healthcare, and finance. Principal component analysis (PCA) is a technique used to reduce the dimensionality of data by transforming it into a set of linearly uncorrelated variables called principal components. These components capture the most significant variance in the data while filtering out less informative details [33]. At the same time, in addition to simple dimensionality reduction, when done correctly, PCA provides feedback on the impact of specific characteristics on variance via the associated eigenvector values that emerge during method implementation.

In this study, the machine learning method PCA is employed to extract temperature indications for luminescent thermometry on recently well-defined material [19]. The hypothesis is that maximizing the temperature-induced variance in the luminescence emission spectra will enhance the sensitivity of the temperature measurement. To implement this, we measure the full emission spectrum of the luminescence probe at various temperatures multiple times (100 times at each T). PCA is then applied to convert the high-dimensional spectral data into a lower-dimensional space, preserving key information from the original data. This research investigates an innovative read-out based on PCA, specifically applied to a tetragonal Eu^3+^-doped Y_2_Mo_3_O_12_ where the entire spectra were used for more accurate and precise temperature measurements instead of selecting specific spectral regions manually. The performance of the PCA method was tested and compared with the results of the traditional luminescence intensity ratio (LIR) approach, highlighting the advantages and effectiveness of PCA in enhancing temperature measurement accuracy and resolution in luminescent thermometry. The LIR approach used here differs significantly from the one previously reported [19] in that it does not entail any computationally demanding procedures such as peak sharpening and is applied to the new dataset used for PCA. Furthermore, the high number of spectra obtained at a single temperature enables a statistical approach to calculating temperature accuracy and uncertainty (temperature resolution).

## 2. Materials and Methods

**Figure 1:**
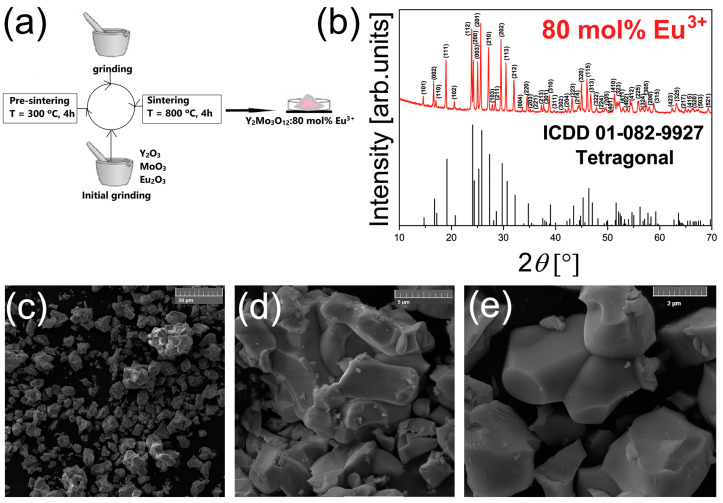
(**a**) Schematic representation of the synthesis procedure; (**b**) XRD measurements of the YMO80Eu sample; (**c**) FE-SEM image of tetragonal YMO80Eu at ×2000 magnification; (**d**) FE-SEM image at ×10,000 magnification; (**e**) FE-SEM image at ×20,000 magnification.

Eu^3+^-doped Y_2_Mo_3_O_12_ was synthesized using the solid-state technique, as previously reported [19]. For the purpose of this study, we synthesized Y_0.4_Eu_1.6_Mo_3_O_12_, a rich in europium content (80 mol%) Y_2_Mo_3_O_12_ that crystallizes in tetragonal form. The stoichiometric amounts of yttrium (III) oxide (Y_2_O_3_, Alfa Aesar, Ward Hill, MA, USA, 99.9%), europium (III) oxide (Eu_2_O_3_, Alfa Aesar, 99.9%), and molybdenum (VI) oxide (MoO_3_, Alfa Aesar, 99.95%) were used as received. The precursors were homogenized by grinding them in an agate mortar with methanol before heating them at 300 °C for 4 h. After cooling to room temperature, the powder was finely ground and subjected to a second annealing at 800 °C in air for 4 h before being left in the furnace to cool to room temperature. The schematic representation of the synthesis procedure is presented in Figure 1a. For luminescence measurements, the resultant pale pink powder was compressed into round pellets without additives at a pressure of 200 MPa.

The crystal structure was investigated by Rigaku SmartLab X-ray diffractometer (XRD, Rigaku, Tokyo, Japan). The diffractometer operated with Cu-K_α1,2_ radiation at a wavelength of λ = 0.1540 nm, under room temperature. The measurements were performed within 2θ range of 10° to 70°, with a step size of 0.02° and a counting rate of 10° per minute. Measured XRD data were analyzed by advanced integrated X-ray powder diffraction suite PDXL2 (Version 2.8.4.0). The morphology was characterized using a TESCAN MIRA3 field emission scanning electron microscope (FE-SEM, Brno, Czech Republic). Prior to imaging, the sample was coated with a thin layer of gold via a standard sputtering technique using a Polaron SC502 system from Fison Instruments, Glasgow, UK. The sample UV–VIS diffuse reflection spectrum (DRS) was recorded with a Shimadzu UV-3600 UV–VIS–NIR spectrophotometer (Kyoto, Japan) in the visible spectral region. BaSO_4_ was used as the reflectance standard. A Fluorolog-3 Model FL3−221 spectrofluorometer system (Horiba JobinYvon, Longjumeau, France) was used to measure the excitation spectrum over the wavelength range of 350 to 580 nm by monitoring the emission at 616 nm. Emission spectra were measured using the Ocean FX UV–VIS Spectrometer (Ocean Insight, Winter Park, FL, USA) with the 405 nm excitation Ocean Insight LED module working in continuous mode and controlled by LDC-1. The MicroOptik heating stage controlled the sample’s temperature. Once the temperature was stabilized, a series of 100 spectra was measured for each temperature. 

## 3. Results and Discussion

### 3.1. Structural and Morphological Characterization

Given the isostructural substitution of Y^3+^ with Eu^3+^ ions in the crystal structure of Y_2_Mo_3_O_12_, it is reasonable to predict that the presence of Eu^3+^ ions would have a minor impact on the structure. However, our previous research revealed that the quantity of incorporated Eu^3+^ ions significantly influences the crystal structure of Y_2_Mo_3_O_12_ [19]. Specifically, the concentration of dopant Eu^3+^ ions causes phase transitions from orthorhombic to tetragonal to monoclinic. In the set of Eu^3+^-doped Y_2_Mo_3_O_12_ samples, luminescence intensity rose with the Eu^3+^ dopant concentration, peaking at 80 mol% Eu^3+^, where the material crystallized in a tetragonal structure. At that concentration, the emission intensity remained stable at 98% of its starting value at 100 °C. Considering all of this, the 80 mol% Eu^3+^-doped Y_2_Mo_3_O_12_ (Y_0.4_Eu_1.6_Mo_3_O_12_) was synthesized as a probe for this study, named the YMO80Eu sample.

To confirm the structure and morphology of the probe, XRD and FE-SEM measurements were performed. Figure 1b presents XRD measurements of the YMO80Eu sample. The XRD pattern is indexed according to the ICDD Card No. 01-082-9927 (Y_2_Mo_3_O_12_), confirming that the probe crystallizes in a tetragonal crystal structure with a P-421m (113) space group. The PDXL2 software (Version 2.8.4.0) confirmed the absence of an amorphous phase, indicating 100% crystallinity, and the structural features acquired from the XRD pattern are given in Ref. [19]. The software used Halder–Wagner and Hall methods to compute crystallite size and lattice strain, resulting in values of 478 (23) Å and 0.06 (5) and 593 (111) Å and 0.08 (4), respectively. The Hall method’s greater sensitivity to both crystallite size and lattice strain results in bigger crystallite size values.

The structure consists of corner-shared [MoO_4_] tetrahedrons and [YO_7_] heptahedrons. Yttrium ions have a stable ionic charge of 3+, making it more likely that europium ions (Eu^3+^) will isostructurally replace Y^3+^ ions. The absence of blue emissions associated with Eu^2^⁺ ions in the luminescence spectrum indicates that the Eu-ions are stabilized in the 3+ oxidation state in this host. The small shift in diffractions to lower values compared to the ICDD Card is caused by the exchange of bigger Eu^3+^ with Y^3+^ ions in the crystal lattice.

Figure 1c–e shows FE-SEM images at magnifications of ×2000, ×10,000, and ×20,000, revealing irregularly shaped agglomerates. These agglomerates are predominantly composed of particles of two microns or greater, with overall agglomeration diameters ranging from several microns to roughly 20 microns. The agglomerates are closely packed as a result of the sintering process, and the powder’s high porosity is due to the existence of agglomerates of diverse sizes. Despite the uneven forms and aggregation, the particles’ surfaces appear quite smooth. These findings indicate that the material, in its present form, is suitable for applications in luminescent thermometry, such as pellet or paint composition. Because the material is in bulk form, structural and morphological characteristics such as crystallite size, crystallinity %, porosity, and particle size have no significant effect on the luminescence intensity ratio and PCA techniques in this study. Additional milling or alternative synthesis procedures could be used in applications that require smaller or more consistent particle sizes, such as biomedicine, microfluidics, thin films, or other specialized fields.

### 3.2. Photoluminescence

**Figure 2:**
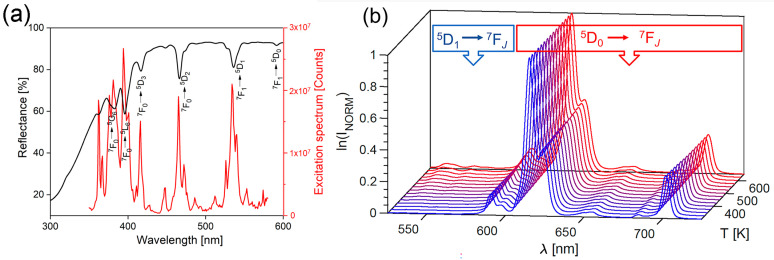
(**a**) Diffuse reflectance spectra (black line with left y) and excitation spectra (red line with right y) of the probe, at room temperature; (**b**) Temperature-dependent normalized emission spectra recorded in the 500–750 nm spectral and 300–650 K temperature range.

The room temperature diffuse reflectance spectrum of the YMO80Eu sample was measured in the 300–600 nm range, revealing distinct absorption bands. The excitation spectrum was recorded from 350 to 580 nm while monitoring the emission at 616 nm, as shown in Figure 2a. The reflectance spectrum exhibits a prominent broad charge transfer band for Eu^3^⁺ in oxygen-rich materials, extending from 340 nm to shorter wavelengths. The absorption features related to Eu^3^ ions from the 4*f*-4*f* transitions occur between 345 and 610 nm. Specifically, the electronic transitions at 381, 396, 416, 466, 537, and 593 nm correspond to the following transitions: ^7^F_0_ → ^5^G_6_, ^7^F_0_ → ^5^L_6_, ^7^F_0_ → ^5^D_3_, ^7^F_0_ → ^5^D_2_, ^7^F_1_ → ^5^D_1_, and ^7^F_1_ → ^5^D_0_, respectively [34]. The excitation spectrum indicates that most absorption peaks align well with the expected excitations of Eu^3+^ in this material.

Figure 2b shows temperature-dependent normalized emission spectra of the YMO80Eu sample recorded between 300 and 650 K upon 405 nm excitation. There are two distinct regions: a ^5^D_0_→^7^F_1–4_ region, which is intense at all temperatures, and a lower wavelength region that corresponds to emission from the ^5^D_1_ level at higher temperatures due to thermalization. The obtained spectra were used for luminescence thermometry with the traditional LIR and PCA methods.

The most significant figures of merit that refer to the quality and potential use of thermometers, namely relative and absolute sensitivity (S_r_ and S_a_), average accuracy (ΔT, the mean value of the difference between the measured and nominal temperature values), and resolution/temperature uncertainty (δT, standard deviation of the Ti(T) distribution), can be calculated using the following equations:

(1) $$S_{a}=\left|\frac{dQ}{dT}\right|;\;S_{r}=\left|\frac{100\%}{Q}\frac{dQ}{dT}\right|$$

(2) $$∆T\left(T\right)=\frac{1}{N_{QC}}\sum_{i=1}^{N_{QC}}\left|{T-T}_{i}\left(T\right)\right|=\left|T-\frac{1}{N_{QC}}\sum_{i=1}^{N_{VAL}}T_{i}\left(T\right)\right|=\left|T-\overset{―}{T_{i}\left(T\right)}\right|,$$

(3) $$\delta T(T)=\sqrt{\frac{1}{N_{QC}-1}\sum_{i=1}^{N_{QC}}{\left(T_{i}\left(T\right)-\overset{―}{T_{i}\left(T\right)}\right)}^{2}}$$

where Q is the thermometric parameter of interest (LIR, PC, lifetime, bandwidth, band shift, etc.). Here, $∆T\left(T\right)$ and δT(T) are calculated experimentally based on the quality control (QC) dataset consisting of N_QC_ spectra measured at fixed temperature T. The average of the temperature readouts based on QC set at a given, fixed, temperature T is denoted as $\overset{―}{T_{i}\left(T\right)}$.

#### 3.2.1. Luminescence Intensity Ratio Readout

LIR readout is the most prominent approach in luminescence thermometry. In Boltzmann-type luminescence thermometry, lanthanide ions with two thermally coupled emitting levels, Level 1 and Level 2, with energy separation ΔE are placed within the wide bandgap of the host material. At low temperatures, where k_B_T<<ΔE, the emission is present only from the lower level since the thermal energy is not sufficient to support the population of the higher energy one. With temperature increase, the upper level becomes more populated due to thermalization, and its emission increases relative to the emission from the lower level. In ratiometric analysis, the ratio of emission intensities from upper and lower levels measures temperature. 

**Figure 3:**
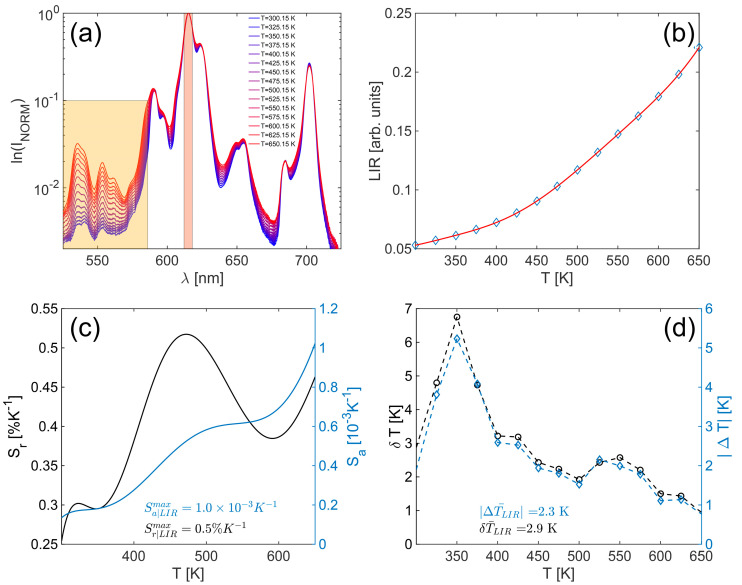
LIR thermometry parameters of YMO80Eu probe: (**a**) Temperature-dependent normalized spectra with designated regions used in LIR method, (**b**) Temperature-dependent LIR, (**c**) absolute and relative sensitivities, (**d**) accuracy and resolution.

When emissions from adjacent thermalized excited levels are utilized, we are then talking about Boltzmann thermometers, as we explored in our previous work [19]. Since in our case the levels were not narrow and strictly defined and since we do not want to apply computationally demanding tasks like peak sharpening, we redefined the LIR regions based on Figure 3a and used a high seventh-degree polynomial to model the data. By using the high-order polynomial, we want to eliminate, as much as possible, the influence of the model choice on the temperature accuracy analysis:

(4) $$LIR(T)=\frac{I_{H}(T)}{I_{L}(T)}=\sum_{i=0}^{7}l_{i}T^{i}$$

Table 1 provides the fit parameters for the LIR polynomial model that were determined using Equation (4).

In Eu^3+^-activated phosphors, two excited levels of interest are ^5^D_0_ and ^5^D_1_. Herein, the temperature-dependent ratio of the integrated emissions in the 525–585 nm (^5^D_1_-level) and 612–618 nm (^5^D_0_-level) range (Figure 3a) was used as the thermometric parameter of interest. As seen in Figure 3a, using the broader range for ^5^D_0_ would lower the LIR sensitivity since outside that range the temperature behavior of normalized spectra is the same as for ^5^D_1_ bands—as temperature increases, the normalized intensities also increase. In Figure 3b, the calculated LIR with appropriate fit is presented. Based on the model from Equation (1), absolute (S_a_—blue line) and relative (S_r_—black line) sensitivities were calculated and presented in Figure 3c. The maximum value for S_a_ is 1.0 × 10^−3^ K^−1^ at 650 K, and that of S_r_ is 0.5% K^−1^ at 472 K. The average accuracy (ΔT–blue diamonds) and resolution (δT–black circles) were calculated based on the mean value and the standard derivation of the temperature readouts at each temperature and are presented in Figure 3d. The average value in the observed temperature range for |ΔT| is 2.3 K, and that of δT is 2.9 K.

#### 3.2.2. Principal Component Analysis Readout

In this approach, normalized spectroscopic data are utilized by treating the intensities at each measured wavelength as features; thus, the number of features equals the number of spectral points, (N_SP_). Each spectrum (Sj) is associated with its corresponding temperature (Tj), yielding a [Sj, Tj] pair that represents one observation. PCA is used to identify a linear combination of all the features in such a way that it maximizes variance. As a result of the PCA method, N_SP_ principal components (PC_i_ $1\leq i\leq N_{SP}$) were identified, with decreasing variance between NS spectra in the input dataset (in our case Ns = 50 spectra × 15 temperatures = 750). Usually, only the first few components provide a significant contribution to the total distribution variance. Often, it is possible to reduce the whole spectrum to just a single principal component (the first one—PC_1_). The value of the PC_1_ can then be used as a thermometric parameter like LIR, band shift, bandwidth, or lifetime.

To obtain principal components one needs to find eigenvalues and their corresponding eigenvectors of the input dataset (N_S_ × N_SP_) covariance matrix (N_SP_ × N_SP_). The resulting eigenvalues represent the variance of the data in the direction of their corresponding eigenvector $\left({PC}_{i}^{\mathrm{coeff}}\right)$; thus, the principal component one $\left({PC}_{1}\right)$ is linked to the highest eigenvalue and consequently all the other principal components to eigenvalues in descending order. To calculate the PC value of any given spectrum, one only needs to find the scalar product between the spectrum and the corresponding eigenvector.

**Figure 4:**
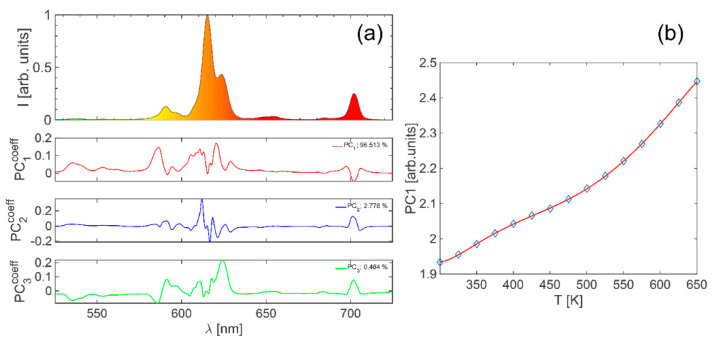
(**a**) Typical spectrum with values for ${PC}_{1}^{coeff}$, ${PC}_{2}^{coeff},$ and ${PC}_{3}^{coeff}$ coefficient vector; (**b**) the mean value of all *PC*_1_ at corresponding nominal T with the polynomial fit.

In Figure 4a, the typical spectrum is presented along with values for ${PC}_{1}^{\mathrm{coeff}}$, ${PC}_{2}^{\mathrm{coeff}}$ and ${PC}_{3}^{coeff}$ coefficient eigenvectors (linear combination coefficients for the features) and their significance—contribution to the total variance. Here, it can be seen that almost all the significance is in *PC*_1_ with 96.5%, with only *PC*_2_ (2.8%) having significance above 1%. The next parametric component, *PC*_3_, has only 0.5% significance. It is also interesting to note the learning component PCA introduces by examining the value of eigenvector ${PC}_{1}^{\mathrm{coeff}}$. Apart from the obvious part of the spectrum contributing to the PC_1_ component (578–590 nm; 603–640 nm), there are also unexpected parts of the spectrum that are important to temperature sensing, like the one in the range from 530 nm to 565 nm, as well as the one from 690 nm to 710 nm where the eigenvector values are not zero: $\left|{PC}_{1}^{\mathrm{coeff}}\right|>0.01$.

(5) $${PC}_{1}(T)=\sum_{i=0}^{7}p_{i}T^{i}$$

Table 2 provides the fit parameters for the PC_1_ polynomial model that were determined using Equation (5). 

**Figure 5:**
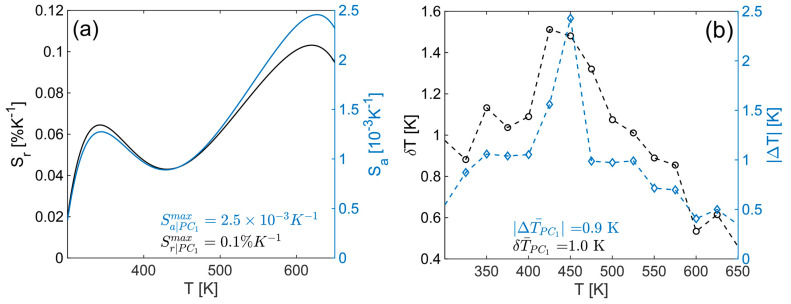
PCA thermometry parameters of YMO80Eu probe: (**a**) Absolute and relative sensitivities; (**b**) accuracy and resolution.

As mentioned, the value of *PC*_1_ can be used as a thermometric parameter, and Figure 4b shows the mean value of all ${PC}_{1}$ from the input at the corresponding nominal temperature (blue diamonds) with the high seventh-order polynomial fit (red line) represented by Equation (5). Based on this, the absolute (S_a_—blue line) and relative (S_r_—black line) sensitivities, presented in Figure 5a, are calculated and have maximum values of 2.5 × 10^−3^ K^−1^ and 0.1%K^−1^, respectively. The values for accuracy (ΔT–blue diamonds) and resolution (δT–black circles), presented in Figure 5b, are calculated based on the distribution of control set (50 spectra not used during PCA calculation) temperature readouts as mean and standard deviation values at each fixed temperature, respectively. The average values in the observed temperature range were found to be 0.9 K for ΔT and 1.0 K for δT.

### 3.3. Comparison Between LIR and PCA Approach

We applied both methods to the same dataset of photoluminescence emission spectra to compare them. Table 3 summarizes the most significant thermometric figures of merit obtained using the LIR and PCA methods. While the LIR method appears five times more sensitive than PCA, PCA stands out with significantly enhanced accuracy and resolution, which is the primary goal. The experimental values derived from temperature readout distributions clearly demonstrate PCA’s superiority, achieving nearly three times better resolution and more than doubling the accuracy of LIR. Moreover, PCA exhibits greater consistency in resolution across the entire temperature range. In PCA, the maximum outlier reaches only 150% of the average, whereas LIR reaches 234%. From a computational standpoint, both methods are straightforward to implement, with no clear advantage for either. However, the improved accuracy and resolution of PCA make it the preferred method for thermometric analysis. This is in line with other previously published applications of PCA [35,36].

**Figure 6:**
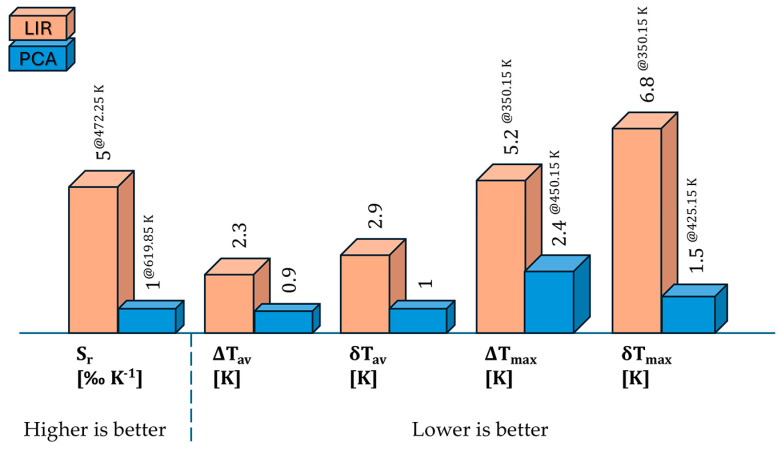
Schematic comparison highlighting the advantages of PCA over the LIR technique based on various figures of merit.

To highlight the advantages of PCA over the LIR technique, Figure 6 depicts a schematic comparison of the figures of merit.

## 4. Conclusions

In this study, we demonstrated the effectiveness of principal component analysis (PCA) as a novel approach for luminescence thermometry using a tetragonal Eu^3^⁺-doped Y_2_Mo_3_O_12_ probe. PCA distinguishes itself from more complex methods by relying solely on basic mathematical operations of addition and multiplication. Applying PCA after calibration is simpler than other techniques, such as multiparametric readout (MLR), which require complicated calculations to process data and identify different LIRs and band positions. PCA streamlines this process significantly. To apply PCA, one simply multiplies the associated principal component vector by the normalized spectrum intensity vector. We discovered that the first principal component carries almost all the influence weight (96.5%), with contributions from full spectra. Our findings highlight that, although the traditional LIR method shows higher sensitivity, PCA significantly enhances both accuracy and resolution, achieving nearly three times better resolution and more than double the accuracy of LIR. This improvement is particularly noteworthy in practical applications where precision in temperature measurement is crucial. Furthermore, PCA demonstrates greater consistency in results across a wider temperature range, showcasing its robustness compared to LIR. Overall, our results advocate for the adoption of PCA in luminescence thermometry, paving the way for more precise and reliable temperature measurements in various scientific and industrial fields.

Looking ahead, the integration of machine learning techniques such as PCA into thermometry presents numerous exciting possibilities. Future research could focus on expanding the range of luminescent materials explored, enhancing the spectral analysis algorithms, and optimizing the machine learning models for real-time applications. Additionally, investigating hybrid approaches that combine PCA with other data analysis techniques may further elevate the sensitivity and reliability of temperature measurements.

Furthermore, the adaptability of these methods in various environments—such as extreme temperatures or high-pressure conditions—could be explored, potentially revolutionizing fields such as aerospace, biomedical applications, and materials science. Ultimately, this study lays the groundwork for future innovations in non-contact thermometry, promising to facilitate more sophisticated, reliable, and user-friendly temperature measurement systems that meet the growing demands of various industries.

## Figures and Tables

**Table 1:** Fit parameters for the LIR polynomial model.

	*l*_0_	*l*_1_ [10^−1^ K^−1^]	*l*_2_ [10^−3^ K^−2^]	*l*_3_ [10^−6^ K^−3^]	*l*_4_ [10^−9^ K^−4^]	*l*_5_ [10^−11^ K^−5^]	*l*_6_ [10^−15^ K^−6^]	*l*_7_ [10^−18^ K^−7^]
Values	10.9575	−1.75941	1.19011	−4.37450	9.43070	−1.19143	8.16924	−2.34591

**Table 2:** Fit parameters for the PC_1_ polynomial model.

	$p_{0}$	$p_{1}$[10^−1^ K^−1^]	$p_{2}$[10^−3^ K^−2^]	$p_{3}$[10^−5^ K^−3^]	$p_{4}$[10^−8^ K^−4^]	$p_{5}$[10^−11^ K^−5^]	$p_{6}$[10^−14^ K^−6^]	$p_{7}$ [10^−18^ K^−7^]
Values	56.4852	−8.06979	5.01888	−1.7076	3.44575	−4.13606	2.74263	−7.76777

**Table 3:** Comparison of PCA and LIR method thermometric figures of merit.

	S_r_ [‰ K^−1^]	ΔT_av_ [K]	δT_av_ [K]	ΔT_max_ [K]	δT_max_ [K]
LIR	5^@472.25 K^	2.3	2.9	5.2 ^@350.15 K^	6.8 ^@350.15 K^
PCA	1^@619.85 K^	0.9	1.0	2.4 ^@450.15 K^	1.5 ^@425.15 K^

## Data Availability

The original contributions presented in the study are included in the article, further inquiries can be directed to the corresponding authors.
